# The Southern Branch of the National Dental Association

**Published:** 1898-04

**Authors:** J. M. Walker


### PROCEEDINGS.

### The Southern Branch of the National Dental Association.

Reported for the DENTAL REGISTER by Mrs. J. M. Walker.

"The Southern Branch of the National Dental Association," as the old "Southern Dental Association" is now called, held its first annual meeting under the new organization, in St. Augustine, Fla., February 22–25, 1898, Dr. E. P. Beadles, Danville, Va., presiding.

The meeting was opened with prayer by the Rev. S. Hamilton Day, of St. Augustine, followed by addresses of welcome from the mayor of the city, the Hon. F. B. Genovar, Dr. J. N. Jones, of Jacksonville, on behalf of the State dental society, and Dr. S. Ewing Smith, on behalf of the dentists and citizens of St. Augustine. Dr. Jones also presented the Association with a beautiful gavel "constructed of Florida woods (palmetto, orange, walnut and pine) by Florida hands, on Florida soil—a genuine *Florida Cracker*."

The President, in his annual address, dwelt upon the experimental character of the meeting—the first under the new regime; the objects had in view by the two associations—the "American" and the "Southern"—in the formation of a National Association; the reasons for continuing the "Southern" along the old lines as far as is consistent with its functions as a branch of the "National," especially in its social features—"the true Southerner" being "a social animal," "created that way." He then defined its present position as a part of, and working in harmony with, the National Dental Association, meeting in the years when the National meets in other sections, as an organization working in its own independent way, but coming more strictly in touch with the National when it comes the turn for that body to meet within the territorial limits of the Southern branch, thus forming one harmonious whole. As "in union there is strength," so will we have greater scientific achievements by a correlation of forces.

He spoke of the cry of "sectionalism," which has been heard in certain quarters, and said "sectionalism" is a much misunderstood thing. We are sectional in that we love our own section best. A man can be a true Floridian, and yet love St. Augustine better than any other city in Florida. So it should be with the different sections of our country. Nobody blames the Westerner because he believes in and boasts of the West. It is not that we of the South love other sections less, but we love our own more. We are willing and glad to clasp hands with our brethren from all sections, in love and charity. We are more than willing to join with them in any and all efforts to promote the welfare of our beloved profession. The President then quoted from and enlarged upon different sections of the Constitution of the National, especially the relations between the branches and the National, and the rights, powers, privileges and duties of the former. He then dwelt upon the unfortunate friction between the National Boards of "Examiners" and of "Faculties," and the means of harmonizing the same; of the appointment of dental surgeons in the army and navy, and also as examiners for life insurance companies. The address concluded by urging the importance of individual effort, if as a body we would make a record to be proud of. Our aim should be perfection. This can only be attained through knowledge. If you carry from here one thought, one idea, that will help you the better to minister to the needs of suffering humanity, your coming will not have been in vain. No sacrifice is too great to be made for the sake of knowledge, and when we have acquired knowledge, it is our duty to impart it to, and use it for the benefit of, our fellow-beings.

